# Supplementary material for: Particulate organic matter as a functional soil component for persistent soil organic carbon
Source: Nat Commun. 2021 Jul 5;12:4115. doi: 10.1038/s41467-021-24192-8 (PMC8257601; doi:10.1038/s41467-021-24192-8)
Supplement: Supplementary file 1 — Supplementary Information [file 41467_2021_24192_MOESM1_ESM.pdf]

Supplementary Material

Particulate organic matter as a functional soil component for persistent soil organic carbon

Correspondence: Kristina Witzgall, kristina.witzgall@tum.de

Supplementary Table 1. Relative chemical composition and alkyl C/O alkyl C ratio of particulate OM fractions after physical fractionation obtained by ^{13}C CP-MAS NMR spectroscopy in the coarse and fine texture.

	Alkyl C (%) (-10)-45 ppm				O/N-alkyl C (%) 45-110 ppm				Aromatic C (%) 110-160 ppm				Carboxyl C (%) 160-220 ppm				Alkyl C:O alkyl C ratio	
Coarse texture	Litter		Control		Litter		Control		Litter		Control		Litter		Control		Litter	Control
fPOM	9.6	± 0.7	14.6	n/a	70.0	± 3.1	55.1	n/a	15.95	± 0.9	22.6	n/a	4.4	± 1.4	7.2	n/a	0.1	0.3
oPOM	9.2	± 0.9	19.9	n/a	70.0	± 2.4	46.0	n/a	17.13	± 0.6	25.7	n/a	3.7	± 0.9	9.5	n/a	0.1	0.4
oPOM$_{small}$	15.8	n/a	18.8	n/a	57.4	n/a	49.2	n/a	20.17	n/a	24.5	n/a	6.6	n/a	7.4	n/a	0.3	0.4
Fine texture	Litter		Control		Litter		Control		Litter		Control		Litter		Control		Litter	Control
fPOM	9.6	± 0.5	16.0	n/a	68.0	± 0.7	59.3	n/a	17.35	± 0.3	19.6	n/a	5.0	± 0.77	5.3	n/a	0.1	0.3
oPOM	9.2	± 0.5	18.4	± 0.5	67.8	± 2.6	51.2	± 0.8	18.3	± 1.0	23.5	± 0.4	4.9	± 1.2	6.9	± 0.3	0.1	0.4
oPOM$_{small}$	17.3	± 0.6	20.8	± 0.3	49.6	± 4.5	49.1	± 2.9	23.7	± 1.9	21.7	± 0.7	9.3	± 2.3	8.3	± 2.2	0.4	0.4

Supplementary Table 2. Relative amounts of carbohydrates, proteins, lignin, lipids, and carbonyls obtained from the application of the molecular mixing model to the ^{13}C CP-MAS NMR spectra of each particulate OM fraction in the coarse and fine texture.

	Relative contents (%)															
	Carbohydrates				Proteins				Lignin				Lipids			
Coarse texture	Litter		Control		Litter		Control		Litter		Control		Litter		Control	
fPOM	56.8	± 2.7	39.3	n/a	8.0	± 0.6	15.4	n/a	32.9	± 2.0	41.6	n/a	2.3	± 0.4	3.7	n/a
oPOM	56.4	± 2.0	30.1	n/a	8.6	± 0.4	19.3	n/a	33.2	± 0.9	45.5	n/a	1.8	± 0.8	5.1	n/a
oPOM$_{small}$	41.7	n/a	31.3	n/a	18.7	n/a	20.0	n/a	34.7	n/a	43.8	n/a	5.0	n/a	4.9	n/a
Fine texture	Litter		Control		Litter		Control		Litter		Control		Litter		Control	
fPOM	53.9	± 0.3	43.6	n/a	10.0	± 0.3	11.3	n/a	34.1	± 0.4	38.8	n/a	2.0	± 0.0	6.3	n/a
oPOM	54.1	± 2.0	33.8	± 0.2	12.3	± 1.5	17.4	± 1.0	32.6	± 0.9	42.4	± 1.5	1.0	± 1.2	6.5	± 0.6
oPOM$_{small}$	33.9	± 4.0	31.3	± 1.9	20.6	± 1.1	21.1	± 0.5	39.7	± 3.2	39.0	± 1.3	5.9	± 0.6	8.6	± 0.6

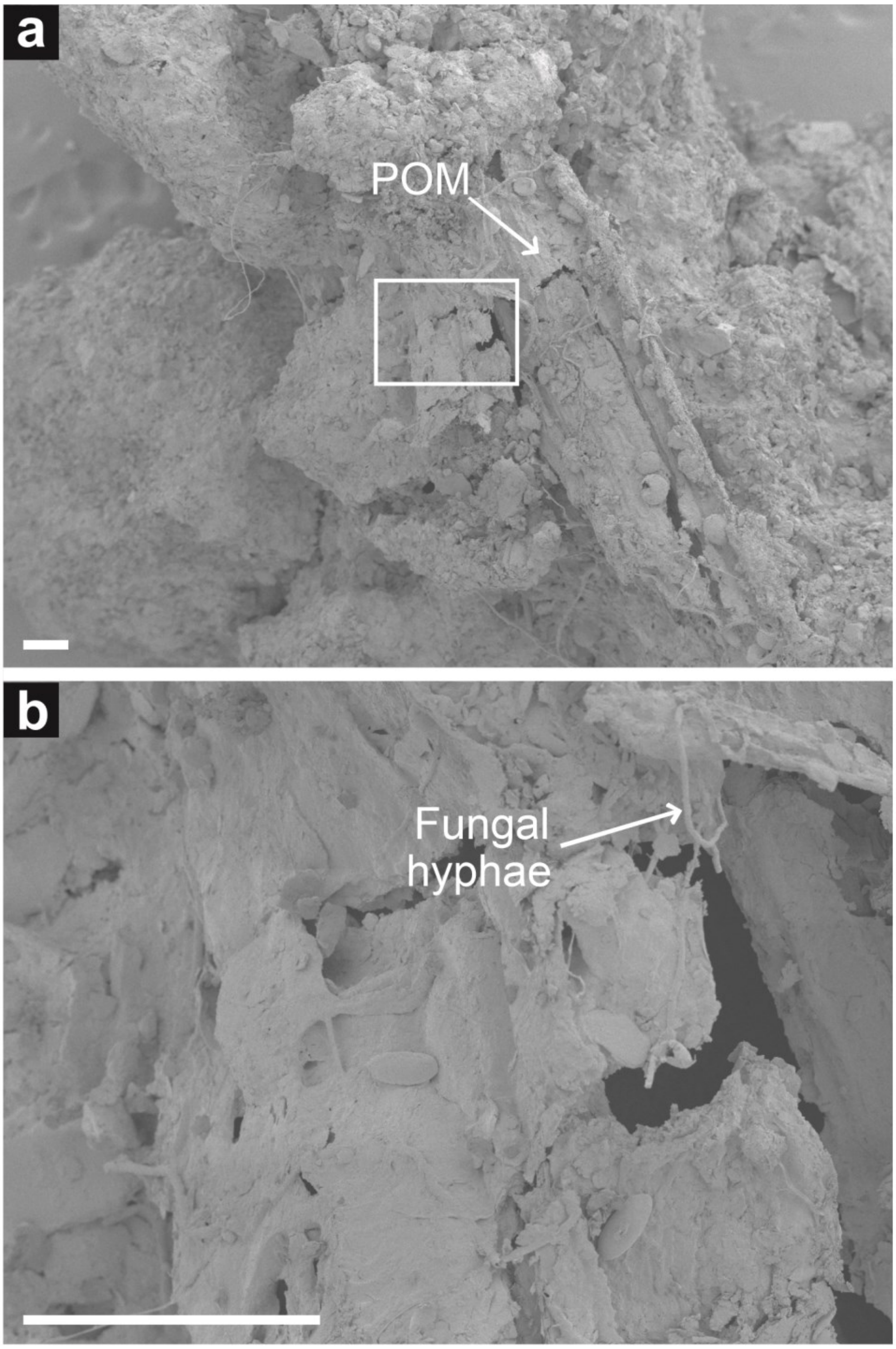

Supplementary Fig. 1. SEM micrographs of POM occluded in a soil microaggregate in intricate connection with organo-mineral associated OM, soil minerals and soil microorganisms. The added litter **a** occluded within a soil aggregate (scale bar = 100 µm) and **b** close-up showing biofilm-like EPS surface coverage and fungal hyphae (scale bar = 100 µm). Similar images were obtained from at least 10 independent locations in each soil texture.

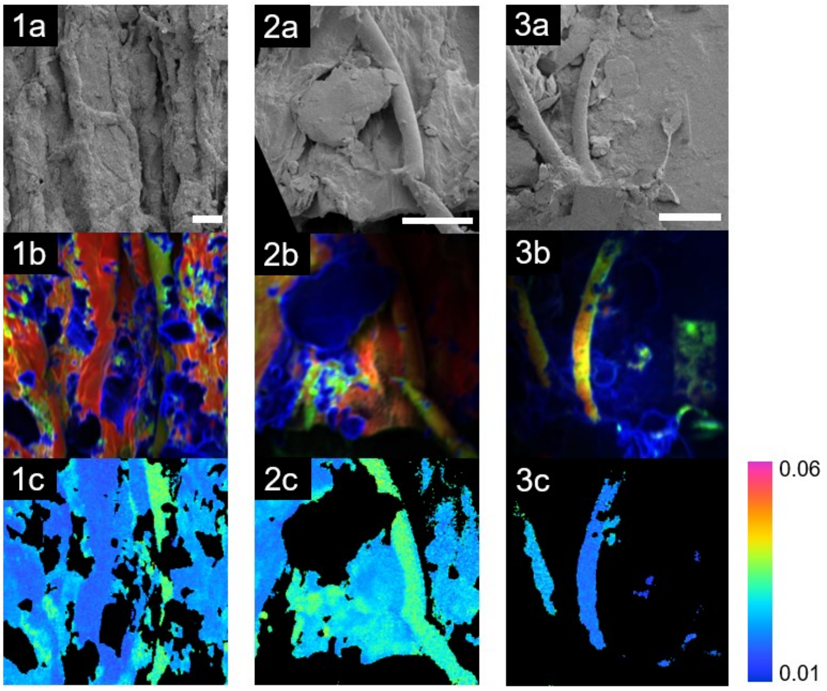

Supplementary Fig. 2. NanoSIMS imaging of selected particulate OM in the coarser textured soil. Each number (**1**-**3**) represents one measurement spot. For each measurement spot, we show **a** SEM micrographs, **b** NanoSIMS composite images displayed as RGB (Red = $^{12}C^{-}$, Green = $^{12}C^{14}N^{-}$ and Blue = $^{16}O^{-}$) and **c** NanoSIMS hue saturation intensity images displaying $^{13}C^{-}$:($^{12}C^{-}$ + $^{13}C^{-}$) ratios ranging from natural abundance (0.01) in blue to high enrichment (0.06) in purple. Scale bars = 10 µm. **2c** shows ^{13}C-enriched fungal hyphae. In each soil texture, at least five NanoSIMS measurements were conducted at independent locations on the POM fragments with similar results.

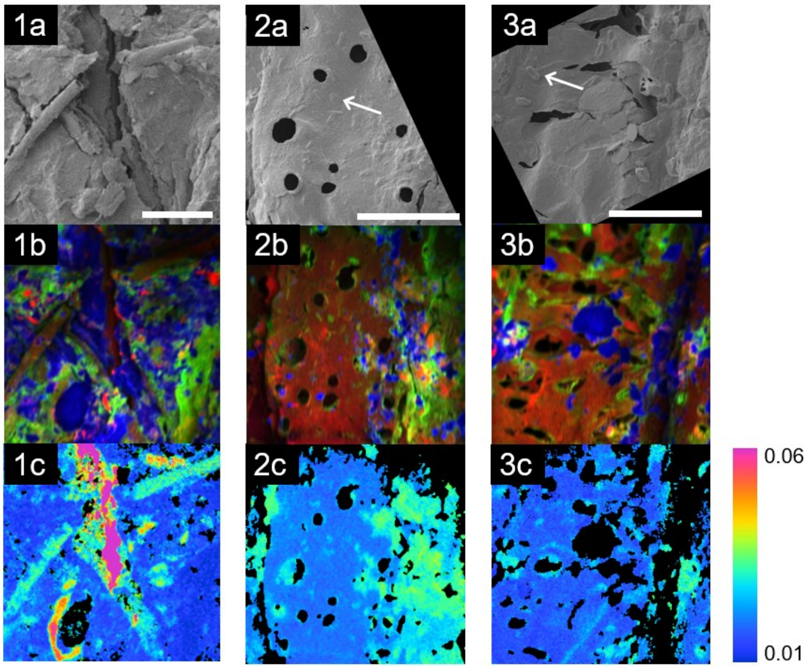

Supplementary Fig. 3. NanoSIMS imaging of selected particulate OM in the finer textured soil. Each number (**1**-**3**) represents one measurement spot. For each measurement spot, we show **a** SEM micrographs, **b** NanoSIMS composite images displayed as RGB (Red = $^{12}C^{-}$, Green = $^{12}C^{14}N^{-}$ and Blue = $^{16}O^{-}$) and **c** NanoSIMS hue saturation intensity images displaying $^{13}C^{-}$:($^{12}C^{-}$ + $^{13}C^{-}$) ratios ranging from natural abundance (0.01) in blue to high enrichment (0.06) in purple. Scale bars = 10 µm. **1c** shows ^{13}C enriched fungal hyphae and **2a**-**c** unicellular microorganisms (presumably bacteria) with high $^{12}C^{14}N^{-}$ contents and ^{13}C enrichment marked with white arrows. Some organisms are also visible in **3a**-**c**. In each soil texture, at least five NanoSIMS measurements were conducted at independent locations on the POM fragments with similar results.

Supplementary Table 3. Basic soil properties of bulk soil of two textures before incubation.

	Texture (%)							
	Clay	Silt	Sand	pH_{CaCl_2}	C (mg g^{-1})	N (mg g^{-1})	C:N	$\delta^{13}C$ (‰ V-PDB)
Finer texture	32.2	53.5	14.4	6.2 ± 0.0	12.2 ± 0.5	1.4 ± 0.0	8.8	-27.4 ± 0.1
Coarser texture	24.1	15.3	60.6	6.2 ± 0.0	4.7 ± 0.5	0.5 ± 0.1	8.8	-27.4 ± 0.1

Supplementary Table 4. Elemental properties of ^{13}C enriched plant litter.

	C (mg g^{-1})	N (mg g^{-1})	C:N	$\delta^{13}C$ (‰ V-PDB)
Plant litter	437.8 ± 3.7	5.5 ± 0.2	80.2	2128 ± 82

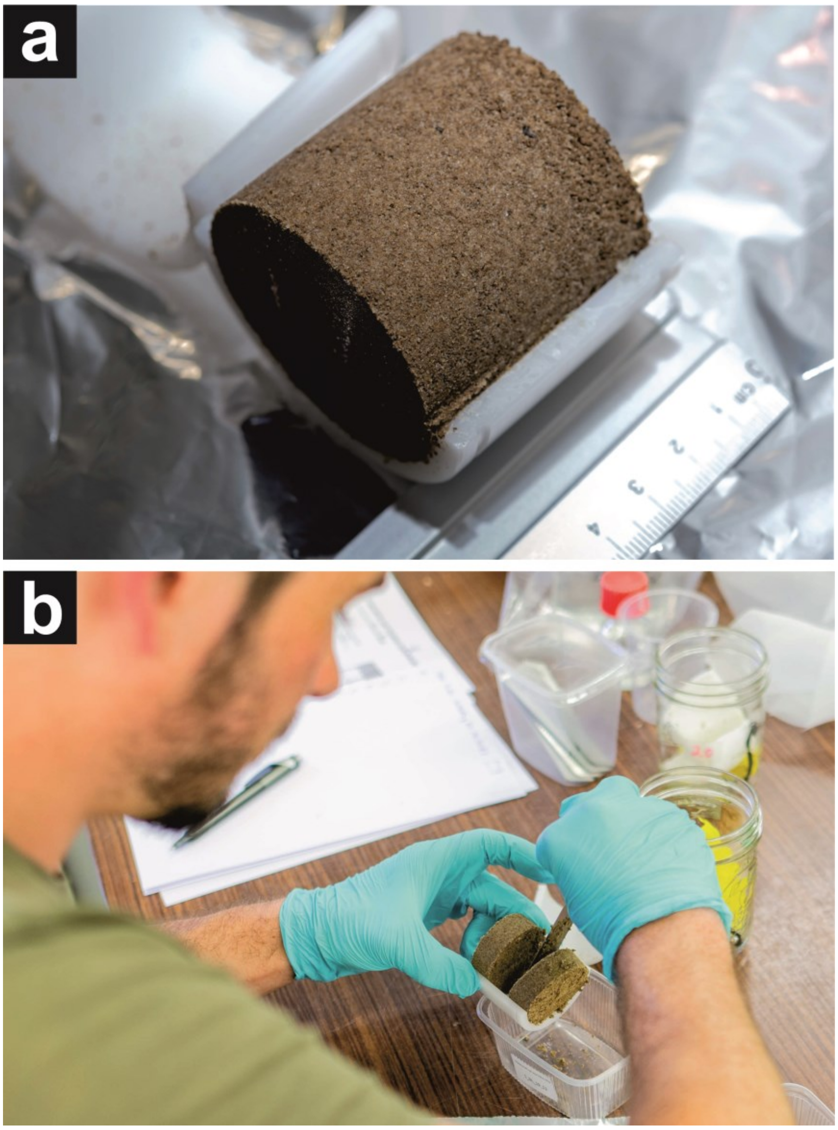

Supplementary Fig. 4. Soil microcosm sampling and depth increments. a Soil microcosms were divided in half after incubation and **b** cut into three depth increments (each 1.67 cm high) using sterile razorblades.

Photo © Carsten W Mueller
