# Supplementary material for: Particulate organic matter as a functional soil component for persistent soil organic carbon
Source: Nat Commun. 2021 Jul 5;12:4115. doi: 10.1038/s41467-021-24192-8 (PMC8257601; doi:10.1038/s41467-021-24192-8)
Supplement: Supplementary file 3 — Reporting Summary [file 41467_2021_24192_MOESM3_ESM.pdf]

nature research

Corresponding author(s): Kristina Witzgall

Last updated by author(s): May 27, 2021

Reporting Summary

Nature Research wishes to improve the reproducibility of the work that we publish. This form provides structure for consistency and transparency in reporting. For further information on Nature Research policies, see our Editorial Policies and the Editorial Policy Checklist.

Statistics

For all statistical analyses, confirm that the following items are present in the figure legend, table legend, main text, or Methods section.

n/a	Confirmed	
☐	☒	The exact sample size (n) for each experimental group/condition, given as a discrete number and unit of measurement
☐	☒	A statement on whether measurements were taken from distinct samples or whether the same sample was measured repeatedly
☐	☒	The statistical test(s) used AND whether they are one- or two-sided<br>*Only common tests should be described solely by name; describe more complex techniques in the Methods section.*
☒	☐	A description of all covariates tested
☐	☒	A description of any assumptions or corrections, such as tests of normality and adjustment for multiple comparisons
☐	☒	A full description of the statistical parameters including central tendency (e.g. means) or other basic estimates (e.g. regression coefficient) AND variation (e.g. standard deviation) or associated estimates of uncertainty (e.g. confidence intervals)
☐	☒	For null hypothesis testing, the test statistic (e.g. F, t, r) with confidence intervals, effect sizes, degrees of freedom and P value noted<br>*Give P values as exact values whenever suitable.*
☒	☐	For Bayesian analysis, information on the choice of priors and Markov chain Monte Carlo settings
☒	☐	For hierarchical and complex designs, identification of the appropriate level for tests and full reporting of outcomes
☒	☐	Estimates of effect sizes (e.g. Cohen's d, Pearson's r), indicating how they were calculated

Our web collection on statistics for biologists contains articles on many of the points above.

Software and code

Policy information about availability of computer code

Data collection	No software was used to collect data.
Data analysis	All statistical testing was carried out in the R statistical environment (Version 1.3.959, © 2009-2020 RStudio, PBC) using agricolae and ggpubr packages. The ImageJ software (Fiji/ImageJ 1.51h; https://imagej.nih.gov/ij) with the OpenMIMS plugin was used to compute ratios for distinct regions of interests and apply dead time and drift corrections in the NanoSIMS measurements.

For manuscripts utilizing custom algorithms or software that are central to the research but not yet described in published literature, software must be made available to editors and reviewers. We strongly encourage code deposition in a community repository (e.g. GitHub). See the Nature Research guidelines for submitting code & software for further information.

Data

Policy information about availability of data

All manuscripts must include a data availability statement. This statement should provide the following information, where applicable:

- Accession codes, unique identifiers, or web links for publicly available datasets
- A list of figures that have associated raw data
- A description of any restrictions on data availability

The data supporting the findings of this study are available on reasonable request from the corresponding author.

Field-specific reporting

Please select the one below that is the best fit for your research. If you are not sure, read the appropriate sections before making your selection.

☐ Life sciences ☐ Behavioural & social sciences ☒ Ecological, evolutionary & environmental sciences

For a reference copy of the document with all sections, see nature.com/documents/nr-reporting-summary-flat.pdf

Ecological, evolutionary & environmental sciences study design

All studies must disclose on these points even when the disclosure is negative.

Study description	We studied the role of soil structure on the fate of litter-derived organic matter. In a 95-day incubation experiment, we investigated how the difference in soil texture influences response variables such as heterotrophic respiration, allocation and stabilization of litter-derived organic matter, and microbial community compositions.<br><br>The experiment was carried out in a full factorial design, with five experimental units (microcosms) for each treatment factor (texture and litter addition). Both treatment factors were two-leveled; coarse and fine soil texture, with and without 13C-labeled litter. During incubation, respiration measurements were carried out on all five replicates. After incubation, the microcosms were divided into three horizontal layers, introducing depth as an additional treatment factor. The analyses following the incubation were carried out on three out of the five replicates. These three replicates were selected based on the content of C and N in each sample compared to the mean of the five replicates.
Research sample	The soil material used in this study origins from an agricultural field (see more detailed information under "Location"). We wanted to represent a common agricultural system and therefore collected soil from an Ap horizon of a Cambisol as it is a typical and widely distributed agricultural soil.
Sampling strategy	In order to capture field variation on site, the soil material was collected from five points along a transect (2 m distance between each point). As the soil was frozen at the time of sampling, we were able to cut out intact blocks of soil at each of the five points. From the blocks, the upper 5 cm densely rooted soil was removed and the material was subsequently collected at depth 5-20 cm. As it is not within the scope of this experiment to account for natural heterogeneity, no independent field samples were collected.<br><br>The collected material was homogenized prior to the experiment to minimize the variability between experimental units. The coarser soil texture was obtained via the addition of quartz sand. In order to yield statistically sound differences between treatments, and in order to rightfully represent the variation between microcosms, the incubation was carried out on a sample size of five replicates per treatment.<br><br>After incubation, great care was taken to avoid contamination during the sampling procedure of incubated microcosms. The microcosms were specifically designed to enable a precise division of individual layers (see Supplementary Fig. 4).
Data collection	David Schubert carried out the incubation and respiration measurements together with Franz Buegger (Q-Box RP1LP Low Range Respiration Package, Qubit Systems, Kingston, Canada; GC/IRMS;Delta Plus, Thermo Fisher, Dreieich, Germany).<br><br>Kristina Witzgall carried out the density fractionation and subsequent elemental analyses were measured together with Franz Buegger (Delta V Advantage, Thermo Fisher, Dreieich, Germany; Euro EA, Eurovector, Milano, Italy). Further, Kristina Witzgall conducted the 13C CP-MAS NMR (Bruker DSX 200, Bruker BioSpin GmbH, Karlsruhe, Germany), and PLFA measurements (GC Agilent HP6890, G1530A, Chemstation, Santa Clara, USA) together with Valérie Pouteau. The SEM and NanoSIMS imagery was done by Kristina Witzgall, Carsten Mueller and Carmen Höschen (Jeol JSM 5900LV, Freising, Germany; Cameca, Gennevilliers, France).
Timing and spatial scale	The incubation experiment lasted from January 15 to April 20, 2018. Gas samples for respiration measurements were collected on 12 days between January 16 and April 19, 2018. The first three measurements were collected on consecutive days (day 2, 3 and 4) to capture short-term changes at initial stages of incubation, followed by nine further measurements (day 8, 10, 15, 23, 31, 44, 65, 80, and 95). The incubation duration was set to be long enough to reach basal respiration, but short enough to not run into problems with nutrient availability for microorganisms. The density fractionation with associated measurements started in June 2018 and the PLFA extractions in October 2018. Lastly, SEM and NanoSIMS imagery were carried out in September 2019. While bulk analytical data is taken from cm scale, imaging data reaches up to µm scale.
Data exclusions	Due to a short-term measurement failure during incubation, two measurement points were excluded for two of the control samples. No further data were excluded from any other analyses.
Reproducibility	A pilot study prior to the experiment was carried out in July 2017. The results of this pilot was used to adopt and improve parameters for the main experiment. The main experiment resembled the overall findings of the preliminary experiment. All relevant basic measurements were carried out (e.g. respiration, CN bulk analyses) and they were nicely resembled in the main experiment reported here. No cost-intense spectroscopic and biomarker analyses were carried out in the preliminary experiment. However, in the experiment reported here, five replicates were used per treatment that showed very good reproducibility. All subsequent attempts of replication were successful.
Randomization	The position of the microcosms during incubation was fully randomized, as well as the order in which respiration measurements were carried out.

Blinding	Blinding does not apply to this study. Knowing the identity of the samples is a prerequisite to avoid cross-contamination during the processing of the samples and to adjust the measurement ranges (IRMS analyses).

Did the study involve field work? ☒ Yes ☐ No

Field work, collection and transport

Field conditions	Soil material used for the experiment was collected on December 11, 2017 (0 °C, no rainfall). The experiment was carried out in a thermal chamber at a constant temperature of 21°C.
Location	The soil material was collected from an agricultural field near Freising, Bavaria, which is part of a research station managed by the Technical University of Munich (Viehhausen, Kranzberg/Freising, Bavaria, 48°23'53.8"N 11°38'39.7"E, 480 m elevation). The area is located within the lower Bavarian upland (mean annual temperature of 7.8 °C and mean annual precipitation of 786 mm). The soil type is a Cambisol (silty clay loam; 32% clay, 53% silt, and 14% sand) with a considerable amount of loess mixed with underlying Neogene sandy sediments.
Access & import/export	All samples were collected in compliance with the regulations set by the research station and no permits were required.
Disturbance	No disturbance was caused by the field collection of the sample material.

Reporting for specific materials, systems and methods

We require information from authors about some types of materials, experimental systems and methods used in many studies. Here, indicate whether each material, system or method listed is relevant to your study. If you are not sure if a list item applies to your research, read the appropriate section before selecting a response.

Materials & experimental systems

n/a	Involved in the study	
☒	☐	Antibodies
☒	☐	Eukaryotic cell lines
☒	☐	Palaeontology and archaeology
☒	☐	Animals and other organisms
☒	☐	Human research participants
☒	☐	Clinical data
☒	☐	Dual use research of concern

Methods

n/a	Involved in the study	
☒	☐	ChIP-seq
☒	☐	Flow cytometry
☒	☐	MRI-based neuroimaging
